# Perception-Induced Effects of Corporate Social Irresponsibility (CSiR) for Stereotypical and Admired Firms

**DOI:** 10.3389/fpsyg.2016.00970

**Published:** 2016-06-24

**Authors:** Seraphim Voliotis, Pavlos A. Vlachos, Olga Epitropaki

**Affiliations:** ALBA Graduate Business School at the American College of GreeceAthens, Greece

**Keywords:** Corporate Social Irresponsibility (CSiR), Corporate Social Responsibility (CSR), stereotype content model, stakeholders, psychology of CS(i)R

## Abstract

How do stakeholders react to Corporate Social Irresponsibility (CSiR)? What are the emotional mechanisms and behavioral outcomes following CSiR perception? The psychology of CSR literature has yet to address these important questions and has largely considered CSR and CSiR as the opposite poles of the same continuum. In contrast, we view CSR and CSiR as distinct constructs and theorize about the cognitive (perceptual), emotional, and behavioral effects of CSiR activity on observers (i.e., primary and secondary stakeholders) building on theories of intergroup perception. Specifically, building on the Stereotype Content Model (SCM; Fiske et al., 2002) and the BIAS map (i.e., Behaviors from Intergroup Affect and Stereotypes; Cuddy et al., 2007)—which extends the SCM by predicting behavioral responses—we make predictions on potential stakeholder reactions to CSiR focusing on two practice-relevant cases: (a) a typical for-profit firm that engages in a CSiR activity, (b) an atypical admired firm that engages in CSiR activity.

## Introduction

While Corporate Social Responsibility (CSR) has received considerable attention (e.g., Sen et al., 2016), the business world has been tarnished with numerous scandals and other irresponsible behaviors, such as environmental pollution or abuses of human rights. These phenomena, conceptualized collectively as Corporate Social Irresponsibility (CSiR), have received comparatively little scholarly attention. Moreover, despite the fact that “individuals act based on perceptions, not objective reality” (Wry, 2009, p. 156), only the emerging microfoundations perspective in CSR (Rupp and Mallory, 2015) and isolated efforts in the CSiR literature (e.g., Lange and Washburn, 2012) take a micro-level view of the effects of CSR and CSiR on the observer of the firm.

Building on recent calls for more research on the micro-level mediating mechanisms that translate stakeholders' CSiR perceptions into outcomes (e.g., Glavas, 2016), we theorize about the cognitive (perceptual), emotional, and behavioral effects of CSiR activity on observers. Thus, we extend the psychology of corporate responsibility literature, which has largely focused on CSR. In particular, we examine the perceptual, emotional, and behavioral effects of CSiR on stakeholders^1^, undertaken by two types of firms: (a) stereotypical for-profit firms that are generally perceived as economically competent but purely self-interested or (b) by admired firms, such as VW, that are perceived as both economically competent and socially responsible (communal). Motivated by the VW software rigging scandal, we consider how such firms may be affected by CSiR activity.

## Theoretical foundations: Firms and the SCM

Our theoretical model examines the effects of stakeholders' perceptions of firms' CSiR activities on their emotions and behaviors toward the firm. Since firms are social evaluation objects (Kervyn et al., 2012), we build our propositions on the psychological study of social relations and, particularly, on the validated, primal, and universal Stereotype Content Model (SCM; Cuddy et al., 2008). The SCM suggests that the perceptual aspect of stereotypes is based on two dimensions, namely *Communality*^2^ and *Competence* (or being *agentic*; Bakan, 1966). Communality is understood as the alignment of the intentions of the perceived to the interests of the perceiver and competence as the ability to bring about desired events (Cuddy et al., 2008). The stereotype content is reduced to a positioning in four clusters within the two dimensional perceptual space of communality and competence: high in both (HCHA)^3^, low in both (LCLA), high communality low competence (HCLA), and low communality high competence (LCHA). Empirical evidence, moreover, positions the stereotyped predominately in the latter two ambivalent clusters. For instance, men (Glick and Fiske, 1999), Jews (Glick, 2002), or professional women (Glick and Fiske, 1996) are primarily perceived as LCHA, while the elderly (Cuddy and Fiske, 2004) or traditional women (Eagly and Mladinic, 1989) are perceived largely are HCLA.

The SCM further posits that perceivers are likely to experience emotions that correspond to each of the four clusters. Specifically, prior research has suggested that *liking* and *respecting* are the affective signatures of communality and competence, respectively (Fiske et al., 1999) and differ in their antecedents: “…liking–disliking is a response reflecting personal interests and preferences, such as fondness (loathing), attachment (dissociation), enjoyment (aversion) […] Respect–disrespect is a response which reflects high regard of and deference to a person” (Wojciszke et al., 2009, p. 39). These affective responses originate from the structural relations between individuals or groups (Glick and Fiske, 2001), as operationalized by their interdependence (competitive vs. cooperative) and by the relative status of the group (Fiske et al., 2002), respectively. For example, individuals respect members of the high-status group but dislike them in competition (Wojciszke et al., 2009), while they disrespect low status-groups but like them in cooperation (Fiske et al., 1999). To summarize, HCHA perceptions generate liking and respecting, LCHA respecting and disliking, HCLA liking and disrespecting, and LCLA disliking and disrespecting^4^.

The SCM is concerned with perceptions of people. However, it has recently been applied to perceptions of firms and brands by stakeholders such as consumers (Aaker et al., 2010, 2012; Kervyn et al., 2012). Although a for-profit firm can, in principle, occupy any cluster, there is evidence that such firms are stereotyped as low in communality and high in competence. By manipulating the “.com” and “org.” heuristics and measuring university and national samples' willingness to buy, Aaker et al. (2010) found such stereotyping, which is not surprising since stereotypes are heuristic categorizations (Fiske and Neuberg, 1990) and the *for-profit* prefix supports a heuristic characterization of low communality. Indeed, although the for-profit company contributes to society by providing employment or meeting consumer needs, societal contribution is not its primary concern (Devinney, 2009). Moreover, the power-ridden, competitive corporate context is likely to elicit perceptions of competence.

Based on the SCM and its extension, the BIAS Map (Cuddy et al., 2007), in what follows we propose the perception-induced behavioral impact of a firm's CSiR on the generic perceiver. We shall first consider the stereotypical LCHA firms and then firms that occupy the HCHA “golden quadrant” (Aaker et al., 2012). We focus on these two clusters because of the aforementioned stereotype and because brand research indicates high scores in the competence scale for all the brands studied (Kervyn et al., 2012 Figure 3)^5^.

## Emotional and behavioral effects of CSiR: The case of the typical for-profit firm

CSiR relates to perceptions of moral transgression and third-party injustice (Lange and Washburn, 2012), both of which powerfully evoke anger. Consequently, CSiR is naturally related to anger, an emotion that expresses moral outrage (Cuddy et al., 2008). Indeed, Grappi et al. (2013) find that companies' ethical and social transgressions engender anger (also, Antonetti and Maklan, 2016)^6^. Although anger does not appear in the SCM, *per se*, one particularly interesting feature of the LCHA cluster is that anger has been found to play a critical role for the observers' behavioral reactions toward the stereotyped. Such stereotype-induced behaviors are predicted by the BIAS map (Cuddy et al., 2007), which has extended the SCM in this respect.

According to the BIAS map, behaviors are represented in two dimensions, intensity and valence. Intensity is characterized as either intense or mild and valence as facilitative or harmful. The BIAS Map predicts, inter alia, that perceptions induce specific combinations of dual behaviors, as mediated by affective responses (i.e., liking and respect). In particular, LCHA perceptions should induce mild facilitation or intense harm, HCHA perceptions will induce mild or intense facilitation, HCLA perceptions will induce intense facilitation or active harm, and LCLA perceptions will induce mild or intense harm. For firms, mild facilitation could amount to simply purchasing the company's products, while discrediting or suing the company could amount to intense harm.

**Figure 1:**
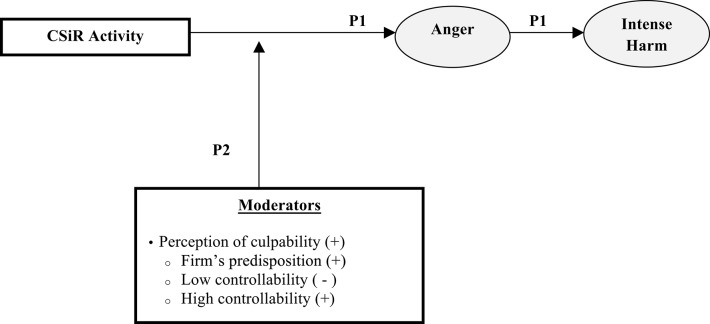
**The impact of CSiR on stakeholders when the firm is perceived as LCHA**.

A stereotypical for-profit company, which is perceived as LCHA, is disliked but respected and should, accordingly, expect to be either mildly facilitated or intensely harmed. However, the theoretically predicted intensely harmful behaviors were only found to occur in the presence of an additional emotion, *anger*, which *fully mediates* the causal link between perception, primary emotion (respect and dislike), and behavior (Cuddy et al., 2007). Thus, we posit (see Figure 1):

*Proposition 1: CSiR activity, when committed by a firm perceived as LCHA, such as a stereotypical for-profit firm, is likely to generate anger which, in turn, is likely to generate intensively harmful behaviors*.

Given the pivotal role of anger in the generation of intensely harmful behaviors, in order to understand the behavioral impact of a CSiR activity by a typical for-profit firm we need to consider the conditions under which CSiR may generate anger. Developing a complete account of such conditions is outside the scope of this article. However, Lange and Washburn (2012) propose, inter alia, that the culpability of the corporation generates attributions of CSiR. We focus here on culpability since it is affected by causal attributions and attribution theory is an important but relatively neglected theoretical mechanism in the micro-CSR literature (Glavas, 2016). In particular, we highlight *controllability* of the causes underlying the CSiR activity, defined as observers' perception that an actor (i.e., the LCHA firm) can affect the causes underlying the activity. Indeed, anger is posited as an attribution of blame (Averill, 1983) and attribution theory predicts that controllability is strongly linked to anger (Weiner, 1985).

Specifically, in the presence of low controllability attributions we expect the effect of CSiR activity on anger to be weakened but, nevertheless, retained. This is because an LCHA firm's intentions are stereotypically perceived as not aligned with observers' interests (Kervyn et al., 2012) and CSiR only serves to confirm these perceptions, which raises doubt concerning the firm's apparent lack of control. On the other hand, when attributions of controllability are relatively higher the effect is straightforward: negative reactions are expected, as the literature on individuals' reactions to acts of injustice predicts (e.g., Miller, 2001). For example, SIEMENS settled a large number of cases of bribery for an estimated €1.3 billion (Patterson, 2009), but could be a “victim” of extortion or competitive pressure within a corrupt institutional field or a “victimizer” who set the corrupt rules (Galang, 2012). We expect the emotional and behavioral reactions of stakeholders to vary substantially depending on the extent of controllability they assign to the firm.

We also expect stakeholders to perceive the firm as more culpable whenever it is perceived as predisposed to irresponsible behavior, that is, “to have a tendency to act in [an irresponsible] way over time” (Lange and Washburn, 2012, p. 306). Thus:

*Proposition 2: Feelings of anger, toward for-profit organizations stereotypically perceived as LCHA that engage in CSiR, are likely to be increased the more the corporation is deemed culpable. Culpability is more likely to be ascribed when the firm is predisposed to irresponsible behavior or when the firm's controllability of the causes is perceived as high, and it is less likely if controllability is perceived as low*.

## Cognitive, emotional, and behavioral effects of CSiR: The case of the admired firm

**Figure 2:**
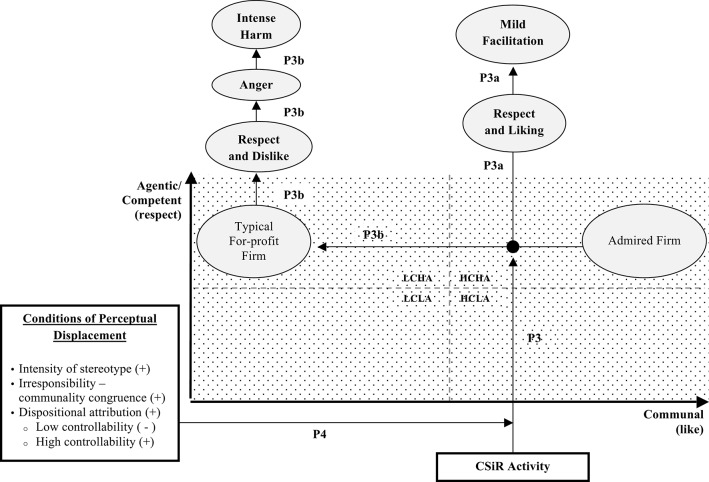
**The impact of CSiR on stakeholders when the firm is perceived as HCHA**.

The second situation of interest concerns admired companies (Kervyn et al., 2012) that occupy the HCHA “golden quadrant” (Aaker et al., 2012), despite the LCHA stereotype. The SCM and the BIAS map, as applied herewith, predict that such companies are expected to be respected *and* liked and to be either mildly or intensely facilitated (see Figure 2). How will perceivers be affected cognitively (perceptually), emotionally, and behaviorally if such firms engage in CSiR?

There are two main possibilities: (a) the firm remains in the same perceptual quadrant (albeit with its reputation dented) or (b) the CSiR activity is potent enough to displace it within the perceptual space to the stereotypical LCHA quadrant. We shall consider both possibilities.

First, since, by supposition, the firm occupies the HCHA quadrant, it is expected to act communally. CSiR is, therefore, not only negative but also unexpected; conditions that increase the salience of the activity. Even if the firm remains in the HCHA cluster, the CSiR activity is likely to dent the liking that it enjoys and, ultimately, the behavioral impact. In particular, since the communality dimension has become salient, behavioral intensity becomes more prominent (Cuddy et al., 2007) and, according to the BIAS map, due to the negativity of the firm's irresponsibility, the facilitative behavioral response is more likely to be mild rather intense. This effect resembles the “insurance-like property” of CSR noted by Godfrey et al. (2009) who found that firms engaging in CSR lost on average (one-third) less capitalization than their counterparts not engaging in CSR. Investing in CSR generates goodwill and moral capital which, in the presence of a negative event, “…should reduce the overall severity of sanctions by encouraging stakeholders to give the firm “the benefit of the doubt”” (Godfrey et al., 2009, p. 428).

If, on the other hand, the firm returns to stereotypically low communality ratings, then the behavioral responses are expected to be quite adverse, as predicted earlier.

*Proposition 3: CSiR activity, when committed by a firm that is perceived as HCHA may either (a) fail to displace the firm from the HCHA quadrant, in which case the firm is likely to experience mild facilitation, mediated by decreased liking and respect, or (b) displace the firm to the stereotypical LCHA quadrant, in which case the firm is likely to experience intensively harmful observer behaviors, mediated by respect, dislike, and anger*.

It is, thus, important to identify conditions that trigger perceptual displacement from HCHA to LCHA. There are several conditions that could drive such displacement and, although, it is outside the scope of this article to theorize about them in detail, we briefly discuss three prominent conditions.

The first condition is the intensity of the for-profit stereotype within the industry: if perceivers firmly believe in it they may assume that the admired company simply reverted to the stereotypically expected behavior of for-profits. For instance, such intensity could depend on geographical locality. Indeed, according to a recent European Commission (2013) non-Europeans are more positive about the overall influence of companies on society. Therefore, it seems that in the EU (vs., for instance, Brazil) the LCHA stereotype is more entrenched, which could result in construing the CSiR activity of an admired company as a manifestation of its non-communal nature.

The second condition concerns the alignment of the act with the underlying dimension of the firm's communality rating. For instance, if a firm, such as VW (Hotten, 2015), is an environmental champion in its industry an environmental infraction, such as VW's software rigging scandal, is likely to affect it more than a taxation impropriety. That is because observers will be more likely to characterize a company as hypocritical when there are domain-specific inconsistencies simply because comparisons are more fluent. Congruity theory (Osgood and Tannenbaum, 1955) predicts that individuals appreciate consistency between what they know and new information. If there is inconsistency—which is easier to diagnose in the case of domain specific communality and irresponsibility, respectively—individuals will try to restore balance by changing attitudes, which in our context may mean displacing the firm to the LCHA quadrant. Our prediction also has implications for the literature that examines the effects of perceptual CSR fit on observer outcomes (e.g., Simmons and Becker-Olsen, 2006), which relates to the extent to which the cause has connections to the firm's core business. Thus, while CSR fit is reported to have positive effects (Simmons and Becker-Olsen, 2006), once a firm engages in CSiR, CSR fit may backfire.

Finally, the third condition relates to the nature of the causal attribution. In particular, if observers perceive the CSiR as a signal of the firm's core then they will attribute it to the firm's disposition which is likely to cause displacement toward the LCHA quadrant in the perceptual space. Specifically, as in proposition 2, if observers view the act as something that is relatively not controlled by the company, such as unrealistically harsh environmental legislation or industry-wide institutional pressures, then the act will not be attributed to the firms' core and perceptual displacement may be avoided. Conversely, if the CSiR is perceived as relatively controllable, it may be attributed to the firm's core and displacement might not be avoided.

*Proposition 4: For-profit organizations perceived as HCHA that engage in CSiR are more likely to be displaced toward the LCHA quadrant if the LCHA stereotype is more entrenched within the industry, if there is congruence between the irresponsible behavior and the firm's communality rating, and if the causes of the irresponsible behavior are attributed to controllable factors*.

## Conclusion

The psychology of CSR literature has primarily focused on companies doing good, largely assuming that CSR and CSiR are the two polar opposites of the same construct. We argue that these are distinct constructs that require separate theoretical examination (for example, the same company might engage in both CSR and CSiR; Kang et al., 2016). That said, we now have some knowledge of how stakeholders react to CSR (Glavas, 2016), yet we know less about stakeholders' reactions to CSiR. Specifically, what are the emotional and behavioral outcomes of CSiR perceptions for stereotypical firms (LCHA) and for admired firms (HCHA)? We give some initial answers to these questions and contribute to the psychology of CSiR by providing a general socio-cognitive model of outcomes—a mediating mechanism between the CSiR activity and its effects—that relies on a validated and parsimonious yet universal model of social perception. Clearly, there is more to follow, for instance: (a) additional moderating factors may be considered, (b) since our model is perceptual, looking at particular classes of observers may yield differential outcomes, and (c) will it be useful for a company to rectify its reputational damage by engaging in CSR following the exposure of its irresponsible behavior?

## Author contributions

SV substantially contributed the conception and design of the work and to the drafting of the paper. PV made substantial contributions to the drafting of the paper and contextualization of the work in the context of CSR. OE made substantial contributions to the drafting of the paper and its potential for operationalization.

### Conflict of interest statement

The authors declare that the research was conducted in the absence of any commercial or financial relationships that could be construed as a potential conflict of interest.

## Footnotes

^1^We develop our propositions without a particular class of stakeholders in mind. Future work could refine our model by examining differentiating nuances between specific observer classes (e.g., employees vs. customers).

^2^The term *warmth* has been originally used in the SCM nomenclature, but for reasons of terminological clarity we use the term *communal* instead (see Abele and Wojciszke, 2007). Indeed, Kervyn et al. (2012, p. 206) note that “…warmth as a trait by itself is easily confused with the perceiver's feelings of warmth.”

^3^We use “A” to denote the second dimension in the acronym, which stands for agentic, since “C” would create obvious ambiguity.

^4^The SCM, in its most sophisticated form, associates the four clusters with the “signature emotions” of admiration, envy, pity, and contempt, respectively (Cuddy et al., 2008). However, we adopt a more rudimentary approach because our focal objects of perception are firms which cannot invoke complex emotions, such as envy, to individuals.

^5^We believe that a large number of not-for-profit organizations, such as governmental organizations, NGOs, and social enterprises would occupy the remaining clusters. We view this as an important and natural extension to our present work.

^6^CSiR may also impact on perceptions of the firm by intensifying the lack of communality. However, since the effect will not change the cluster in which the firm is positioned and since our predictions are cluster-based we ignore such effects here.
